# Meat and bone meal stimulates microbial diversity and suppresses plant pathogens in asparagus straw composting

**DOI:** 10.3389/fmicb.2022.953783

**Published:** 2022-09-20

**Authors:** Xinxin Liu, Xiaoxiao Li, Yinfeng Hua, Aki Sinkkonen, Martin Romantschuk, Yanfang Lv, Qian Wu, Nan Hui

**Affiliations:** ^1^School of Agriculture and Biology, Shanghai Jiao Tong University, Shanghai, China; ^2^Shanghai Yangtze River Delta Eco-Environmental Change and Management Observation and Research Station, Ministry of Science and Technology, Ministry of Education, Shanghai, China; ^3^Shanghai Urban Forest Ecosystem Research Station, National Forestry and Grassland Administration, Shanghai, China; ^4^Shanghai Pudong Development (Group) CO., Ltd., Shanghai, China; ^5^Department of Garden Technologies, Horticulture Technologies, Natural Resources Institute Finland, Helsinki, Finland; ^6^Faculty of Biological and Environmental Science, University of Helsinki, Lahti, Finland; ^7^Food Safety Key Lab of Liaoning Province, College of Food Science and Engineering, Bohai University, Jinzhou, China; ^8^Boda Environmental Protection Co., Ltd., Yixing, China

**Keywords:** meat and bone meal, compost, microbial community diversity, plant pathogen suppress, straw

## Abstract

Meat and bone meal (MBM), as slaughterhouse waste, is a potential biostimulating agent, but its efficiency and reliability in composting are largely unknown. To access the MBM application to the composting process of asparagus straw rice, we followed the composting process for 60 days in 220-L composters and another 180 days in 20-L buckets in treatments applied with MBM or urea. The microbial succession was investigated by high-throughput sequencing. Compared with urea treatments, MBM addition stabilized pH and extended the thermophilic phase for 7 days. The germination index of MBM treatments was 24.76% higher than that of urea treatments. MBM also promoted higher microbial diversity and shifted community compositions. Organic matter and pH were the most significant factors that influence the bacterial and fungal community structure. At the genus level, MBM enriched relative abundances of organic matter-degrading bacteria (*Alterococcus*) and lignocellulose-degrading fungi (*Trichoderma*), as well as lignocellulolytic enzyme activities. Notably, MBM addition decreased sum abundances of plant pathogenic fungi of *Phaeoacremonium, Acremonium*, and *Geosmithia* from 17.27 to 0.11%. This study demonstrated the potential of MBM as an effective additive in asparagus straw composting, thus providing insights into the development of new industrial aerobic fermentation.

## Introduction

Meat and bone meal (MBM) has been utilized for many years as valuable source of proteins and minerals in the livestock feed. As a consequence of transmissive spongiform encephalopathies, the utilization of MBM for animal feed was banned in 2000 in the European Union (European Commission, 2000). The new legal situation made it necessary to seek for alternative disposal strategies for MBM. In fact, MBM contains considerable nutritive elements including N, P, and Ca, which are fundamental substrates for aerobic composting (Jeng et al., 2007; Ylivainio et al., 2008). MBM also contains diverse organic compounds, such as amino acids, which may optimize the C/N ratio, enhance microbial activity, influence microbial community succession during composting, and ultimately affect the composting process. MBM has lower risk of antibiotic resistance gene spreading compare to animal manure (Heuer et al., 2011; Qian et al., 2018). We have previously found that MBM can stabilize pH in bioremediation, which may help compost inhibit pathogenic microbes, since pH increase resulting from compost amendment showed a consistent relationship with the suppression of plant diseases; yet, the effects of MBM in composting have been poorly characterized (Dang et al., 2021).

The huge and increasing asparagus (*Asparagus officinalis* L.) cultivation poses a pressure on agriculture waste treatment (Schwarz and Mathijs, 2017; Pegiou et al., 2020). Asparagus straw composting is a more environmental-friendly solution than discarding and burning as these methods may induce disease spreading and air pollution (Zhao et al., 2017; Chen et al., 2019). Asparagus straw contains a large amount of recalcitrant substances (e.g., cellulose, hemicelluloses, and lignin), which are difficult to be utilized by microorganisms. Consequently, it becomes a major bottleneck to efficient composting of asparagus straw. Maintaining high microbial diversity and activity is beneficial for asparagus straw biomass biodegradation since diverse microorganisms secrete a variety of enzymes to decompose lignocellulose (Li et al., 2016). MBM, a low-cost by-product, is rich in complex organic compounds, which provide diversified substances for microbial incubation (Jeng et al., 2007). MBM contains more slowly soluble than readily available N, P, and K, and it is suitable for stimulation of microbial activity (Mondini et al., 2008). MBM has previously been shown to increase oil degradation in soils when compared with natural attenuation while maintaining a proper soil pH to ensure microbial activities (Liu et al., 2019). Therefore, it is reasonable to predict that MBM can promote biodiversity in the composting process and stimulate lignocellulose degradation. Furthermore, some studies showed that adding meat and bone meal to soil led to suppressed soilborne plant diseases (Tenuta and Lazarovits, 2004; Lazarovits, 2010). Yet, the potential of MBM in suppressing plant pathogens in compost is not determined.

Microbial communities and their succession are directly related to composting duration and quality. Several studies have focused on promotion of composting efficiency by screening microbial communities in early stages of composting, including mesophilic and thermophilic phases (Shi et al., 2018; Chen et al., 2021a). However, the curing phase has drawn much less attention, which leaves the final compost quality data, for example, abundance of pathogens, unavailable.

To investigate the potential of MBM as an additive in asparagus straw composting and the quality of the end compost product, we conducted a small-scale asparagus straw composting experiment in composters (220 L, 12 units) to compare the effects of MBM and urea on the composting process by assessing their influence on organic matter (OM) degradation, compost maturity, the microbial succession across all composting phases, and finally the quality of the finished compost. Furthermore, we propose MBM as a novel additive to achieve rapid composting and high-quality product of compost from asparagus straw waste. We hypothesize that the MBM treatment results in a greater microbial diversity than the urea treatment since MBM contains complex organic substrates to be utilized by a greater variety of microorganisms.

## Methods and materials

### Experiment setup

The 240-days composting experiment was established at an indoor workshop to simulate high-temperature composting of asparagus straw. The workshop was air-conditioned with a constant temperature of 23°C. Asparagus straw samples were taken from an asparagus greenhouse facility, near the county of Gaocheng in China. The asparagus straw samples were cut into pieces <8 cm in length. Meat and bone meal (meat/bone ratio of about 2:1, dry weight), in the dry powder form with <5% moisture, was obtained from Nanfang Co. Ltd. Urea (Anhui Liuguo Chemical Co. Ltd.) was selected as the nitrogen source in the control treatment because of its potential as a stable and effective source in promoting microbial activity, as mentioned in several composting asparagus straw studies (Wei et al., 2018, 2019; Feng et al., 2021).

The experiment was set up in 12 units of Biolan Quick Composter (Biolan, Finland) of 220 L capacity and built with a freon-free polyurethane insulation material and adjustable ventilation. All experiments started on the same day of October 10, 2019. The composters were thoroughly cleaned and sterilized by using 70% ethanol before the experiment. Each composter was loaded with 180 L of asparagus straw weighing ~15 kg. Composters 1–3 were designated as controls in where 2.5% urea (w/w) was added. This achieved a C/N ratio at about 25:1—the same ratio for the industrial-scale asparagus straw composting in Wuxi region. Composters 4–12 were designated as MBM treatments. To test the best MBM amendment rate to composting, we established three groups of treatments containing 2.5, 12.5, and 25 % MBM (w/w), respectively. The 2.3 kg MBM treatment (12.5%, w/w) is comparable with control urea treatment because both cases have a similar C/N ratio of 25:1.

To avoid the possible contaminants that tap water may introduce to the system, Milli-Q water was used throughout the study. Urea was dissolved in Milli-Q water and mixed with asparagus straw using an electronic mixer (L775W, Dingguo, China). MBM and asparagus straw were also mixed. The initial moisture content was adjusted to 60% by adding 10 L Milli-Q water. The ventilation valves were adjusted to 50% in all composters. To homogenize the materials and increase oxygen in the composting, we mixed the asparagus straw using barb-tipped sticks (Biolan, Finland) every day once until the temperature reached 55°C. Mixing stopped on day 12 for composters 4, 5, 6, and 12 because their maximum temperature did not exceed 51.3°C. For composters 1–3 and 7–11, mixings were carried out on days 14 and 30, but the mixing did not result in elevation of temperature. The composts were transferred from composters to 20-L buckets on day 60, and the top-opened buckets were kept at the same workshop for additional 180 days (240 days in total).

### Compost sampling and analyses

The composters were sampled at a depth of 25 cm using a soil push corer (7 cm diameter) in the middle of the composter on days 7, 14, and 30, respectively. The bucket samples were collected at a depth of 10 cm using plastic spoons in the middle of the bucket on days 60, 120, and 240. At each sampling time, five subsamples of the same composter/bucket were merged as one sample. The minimum distance between subsamples was 15 cm. The samples were stored in polyethylene bags at −20°C before biochemical analysis. The samples were thawed at +4°C and sieved using a 2-mm mesh, to remove asparagus straw samples and large particles.

The pH and electrical conductivity (EC) values of leach liquor of composts (composting material:distilled water = 1:10, w/v) were measured using a pH meter (PHS-25, Shanghai Inesa instrument Co. Ltd., China) and a conductivity meter (DDS-307, Shanghai Inesa instrument Co. Ltd., China), respectively. The dry weight was measured from the weight loss of ~5 g samples incubated at 105°C for 16 h, followed by sample treatment at 550°C for 4 h in a muffle furnace, from which the organic matter (OM) was measured. Total potassium (TK) and total phosphorus (TP) were determined colorimetrically after wet digestion with H_2_SO_4_ and HClO_4_, respectively (Parkinson and Allen, 1975). Soil total nitrogen (TN) was determined by following the Kjeldahl digestion procedure (Tabatabai and and Bremner, 1972). Ammonium nitrogen (NH${}_{4}^{+}$-N) and nitrate nitrogen (NO${}_{3}^{-}$-N) contents were determined by using a semi-automatic elemental analyzer (PE2400, PerkinElmer, United states) after extracted by potassium chloride and calcium chloride, respectively. Absorbances were determined at 465 nm (E4) and 665 nm (E6) for 0.05 N NaHCO_3_ solutions containing 0.02% (w/v) compost samples in accordance with Kim et al. (2008). Germination indices (GI) of the compost were determined using Chinese cabbage (*Brassica parachinensis*) seeds, as described earlier by Yang et al. (2020). Lignin peroxidase activity was estimated by using the method of Tien and Kirk (1983). The assay was based on the oxidation of veratryl alcohol (3, 4-dimethoxybenzyl alcohol) to veratraldehyde in the presence of H_2_O_2_, and the increase in absorbance at 310 nm was monitored. The reaction mixture contained 0.25 mL of compost extracts, 0.25 mL of 1 mM veratryl alcohol, 0.2 mM H_2_O_2_, and 0.5 mL of 0.1 M citrate buffer. The cellulase activity was quantified from the reducing sugars generated from incubation of 1 ml compost extracts with 0.1 M acetate buffer (pH 6.5) for 1 h at 37°C and the subsequent reaction with the addition of 3,5-dinitrosalicylic acid solution as per the standard method, using carboxy methyl cellulose as the substrate (Miller, 1959). All measurements were taken in triplicate.

### DNA extraction and PCR amplifications

Total DNA of each sample was extracted from 5 g sieved compost debris using a DNeasy PowerMax Soil Kit (QIAGEN Inc.) and quantified using a PicoGreen DNA Assay Kit (Invitrogen) following the manufacturer's protocol. PCR amplification of the V3–V4 regions of the bacterial 16S rRNA genes was performed using the forward primer 338F 5′-ACTCCTACGGGAGGCAGCA-3′ and the reverse primer 806R 5′-GGACTACHVGGGTWTCTAAT-3′(Suzuki and Giovannoni, 1996; Caporaso et al., 2012). For fungal internal transcribed spacer (ITS) amplification, a similar approach was employed using the forward primer F-ITS7 5′-GTGARTCATCGAATCTTTG-3′ and the reverse primer ITS4 5′-TCCTCCGCTTATTGATATGC-3′ (White et al., 1990; Ihrmark et al., 2012). The quality of the extracted DNA and PCR products was checked by agarose gel (1.5%) electrophoresis. After the individual quantification step, amplicons were pooled in equal amounts, and paired-end 2 × 300-bp sequencing was performed using the Illumina MiSeq Platform with MiSeq Reagent Kit v3 at the Analytical Center, Shanghai Jiao Tong University. Negative controls were included throughout the PCR and sequencing steps. The paired fastq files are available in the Sequence Read Archive at the National Center for Biotechnology Information (www.ncbi.nlm.nih.gov) under accession numbers SAMN20518185–SAMN20518220 for bacteria and SAMN20518136–SAMN20518171 for fungi.

### Quantitative PCR (qPCR) assays

The quantitative PCR technique was used to analyze ribosomal genes of samples from each sampling point (7^th^, 14^th^, 30^th^, 60^th^, 120^th^, and 240^th^). The qPCR assay standards were a series of solution dilutions of *Cupriavidus necator* pJP4 (DSM 4058) for bacteria and *Saccharomyces cerevisiae* (DSMZ 1334) for fungi. Amplification and detection of fungal DNA were performed by using the F-ITS7 and ITS4 primers, and 338F and 806R primers were used for bacterial DNA amplification. The same primers were also used for MiSeq amplicon generation. The total bacterial 16S rRNA genes and fungal ITS genes were quantified using a LightCycler 96 qPCR machine (Roche Life Science). Amplification was conducted using 2.0 μL of diluted DNA (dilution of 1:100), 10 μL of 2X DYNAMO Master Mix, 1 μL of each primer (10 μM), and 6 μl of sterile distilled water. The thermal cycling conditions followed were similar to those described by Yan et al. (2020). A negative control (ddH_2_O) was included, and all samples were run in triplicate.

### Bioinformatics

The sequence data of both partial bacterial 16S rRNA genes and fungal ITS amplicons were analyzed using mothur V.1.42.1 (Schloss et al., 2009). The sequence analysis followed the MiSeq SOP (Kozich et al., 2013). In brief, bacterial sequences were aligned against SILVA (Quast et al., 2012), preclustered to remove erroneous reads (Huse et al., 2010), and screened for chimeras using the UCHIME algorithm (Edgar et al., 2011), and non-chimeric sequences were assigned to taxa using the Naïve Bayesian Classifier (Wang et al., 2007) against the RDP training set (version 10). Non-target sequences (mitochondria, chloroplast, and Archaea) were removed prior to calculating a pairwise distance matrix. Sequences were clustered to OTUs at 97% similarity using nearest neighbor (single-linkage) joining, which conservatively assigns sequences to OTUs.

In fungal ITS sequence analysis, to permit pairwise alignment of fungal ITS sequences to calculate a pairwise distance matrix, we removed a forward primer sequence (ITS1F), omitted all fungal ITS sequences that were <250 bp in length, and truncated the remaining sequences to the first 250 bp (Hui et al., 2017). For quality control, the sequences that contained ambiguous (N) bases and homopolymers longer than 13 nucleotides were screened out. The remaining sequences were preclustered to allow for up to 2-bp difference to remove potential sequencing errors before the identification of the chimeric sequences using the VSEARCH algorithm. After removing the chimeric sequences, the unique sequences were classified using the mothur-formatted UNITE taxonomy reference database, using the default bootstrapping algorithm (cutoff value: 80%). Only fungal sequences were kept and assigned into OTUs by classification using FASTA as the split method, based on nearest neighbor clustering at 97% similarity. Fungal OTUs were assigned to the functional guild using the FUNGuild database with “highly probable” confidence (Nguyen et al., 2016).

In both bacterial and fungal data sets, low-abundance OTUs (≤ 1 sequences across all 36 samples; 3.3% of bacterial and 1.2 % of fungal total reads) were removed as they may be PCR or sequencing artifacts (Tedersoo et al., 2010; Brown et al., 2015; Oliver et al., 2015). We estimated richness and diversity metrics for bacterial and fungal communities in mothur (version 1.47.0). Observed OTU richness (S_obs_), the complement of Simpson's diversity (1/D: 1/∑p${}_{\text{i}}^{2}$), and Simpson's evenness (E_D_: 1/∑p${}_{\text{i}}^{2}$/S), with p_i_ representing the frequency of each OTU within a sample, were iteratively calculated and rarefied by the “subsample” function.

### Statistical analysis

The treatments with MBM 2.3 kg was chosen for the continued comparisons with urea treatments as they performed the best in the temperature increase. All statistical analyses were performed in R (version 3.2.1, R Development Core Team) using various packages. The physicochemical properties of samples were carried out in triplicate, and the mean values with standard deviation are presented. One-way analysis of variance (ANOVA) to compare the mean values for different levels of sampling time (*P* < 0.05) during composting. Non-metric multidimensional scaling (NMDS) analyses were performed for bacterial and fungal data sets using the vegan package to visualize the bacterial and fungal communities. The community structure responds to MBM, and urea additions were determined using the permutation test (envfit function in vegan, permutations = 99,999) (Oksanen et al., 2020). The data were additionally subjected to two-way ANOVA, with treatment and composting time as factors, followed by Tukey's significant difference as a *post hoc* test (HSD, honestly significant difference at the 95% confidence interval).

## Results and discussion

### Effect of meat and bone meal on compost properties

**Figure 1:**
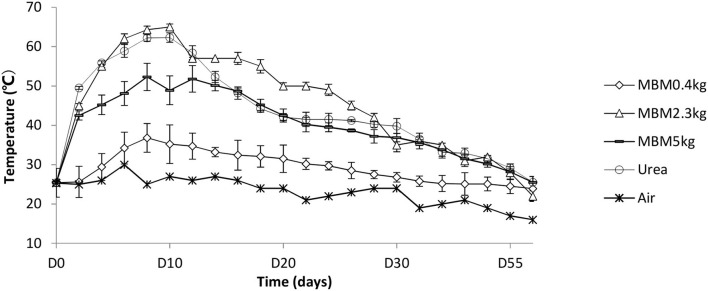
Temperature dynamics during composting experiment. MBM, asparagus straw with additive meat and bone meal; urea, asparagus straw with additive urea.

The dynamics of temperature during the rest of the trial were typical for the biowaste compost (Figure 1). Temperature is a critical factor for indicating the composting reaction rate because of its effect on the microbial metabolic rate and population structure. Studies indicated that the maximum thermophilic composting activity may be achieved at temperatures in the range of 50–60°C Tang et al., 2007; Feng et al., 2021. In addition, high temperature is critical for pathogen removal (Awasthi et al., 2019). Low temperature in 25 and 2.5 % MBM is likely due to the improper C/N ratio, which is an essential condition for microbial activity and temperature rise in straw composting (Jusoh et al., 2013). The temperature of urea-treated compost remained over 50°C from days 2 to 14. The temperature of 12.5% MBM treatment was 65.7°C, while the highest temperature in the urea treatment was 62.3°C. Urea is an organic additive often requiring a temperature of over 50°C (Veijalainen et al., 2015; Feng et al., 2021). We observed the duration of high temperature (higher than 50°C) in MBM treatment was 7 days, which was longer than that in urea treatment. MBM has been reported to promote the size and activity of soil microorganisms (Mondini et al., 2008). Organic additives have been shown to stimulate the microbial activity and lead a longer duration of the thermophilic phase than regular composting (Gabhane et al., 2012). The observed higher temperature could be explained that MBM promoted growth of microbes, leading to a boost in microbial metabolism. The slower start but extended duration of the thermophilic phase in MBM treatment could be due to the constant supply of nutrients by MBM to the composting matrix as much of the N content in MBM is organically bounded (Möller, 2015). In addition, such higher and longer thermophilic phase may benefit pathogen suppression in compost products as most of the plant pathogen microbes cannot survive peak compost temperatures of 60–62°C (Lodha et al., 2002). There was a general gradual decline in temperature in the piles as composting progressed after day 15. The temperatures of all composters approximated to the ambient temperature in 60 days. In this study, the temperatures of 25 and 2.5 % MBM treatments did not reach 50°C, indicating inefficient metabolic rates, so only the 12.5% MBM treatment was used for further investigation.

**Figure 2:**
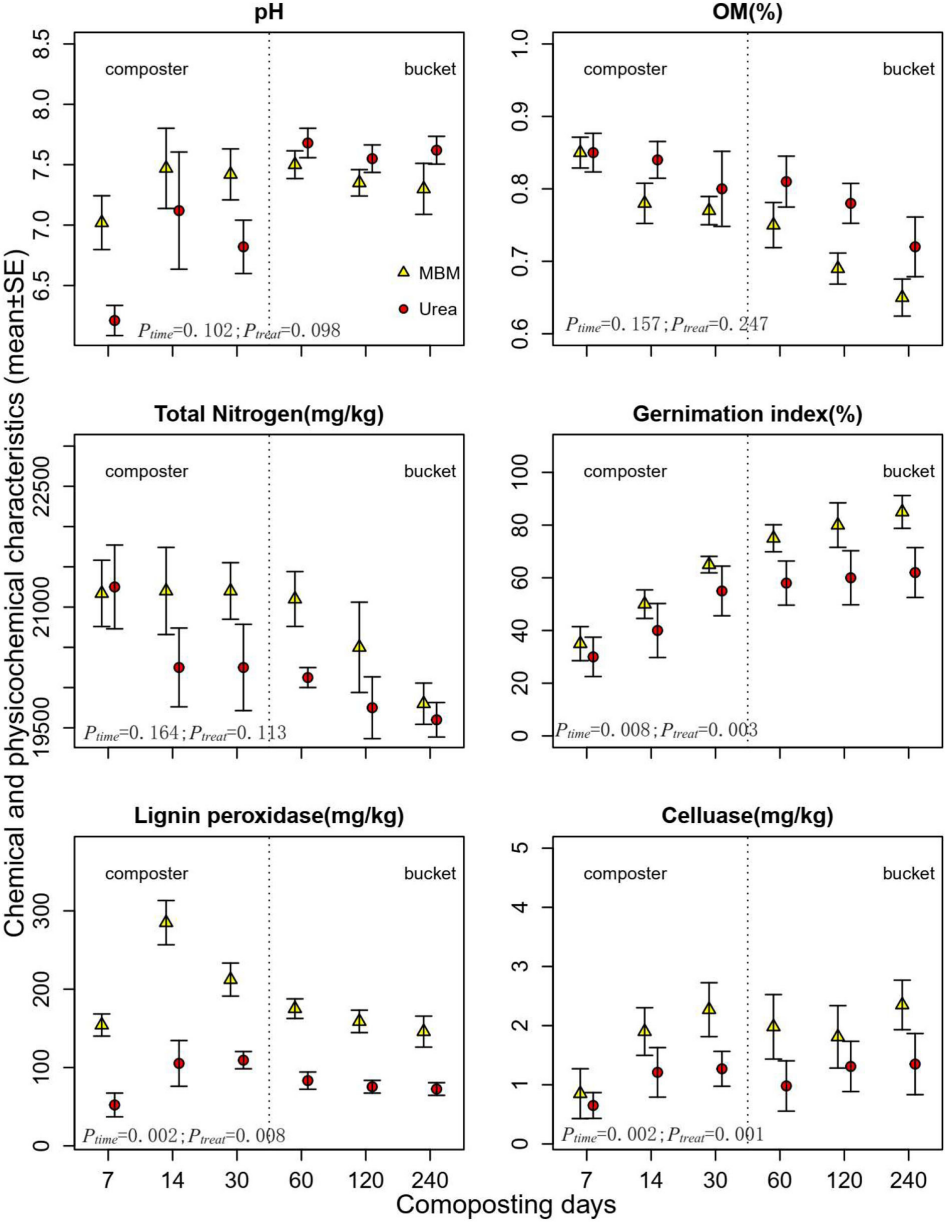
Dynamics of pH, organic matter content (OM%), germination index, total nitrogen, lignin peroxidase, cellulase, and composting process. MBM, asparagus straw with additive meat and bone meal; urea; asparagus straw with additive urea. Error bars are standard deviations of the means of triplicates.

In composters, the pH of urea treatment increased from initial 6.21 to 7.11 and then decreased to 6.74, while the MBM-treated compost reached the highest pH on day 14 (pH ranged from 7.08 to 7.45). Throughout the study period, in the MBM treatment, the pH level had not increased above eight in the compost (Figure 2). During the 240 days of study, MBM did not lead to a rapid pH fluctuation and retained pH more stable in the neutral range (pH 7.0–7.5), which is favorable for organic matter biodegradation. A suitable pH in the neutral to weakly alkaline range is essential for microbial growth, and too high or low pH may harm the activity of lignocellulase and microbial growth because it creates an uncongenial condition for lignocellulase activity and for the growth of lignocellulose decomposers and inhibits microbial activities during composting (Diaz and Savage, 2007; Partanen et al., 2010). At the latter stage of the composting process in the bucket, the value increased until the pH of the end product reached a maximum value (pH 7.5). Unlike the volatile pH in the urea treatments, MBM treatments held a stable pH in the range of 7.05–7.48. In the composting process, the microorganisms initially produced organic acids (acetic acid, butyric acid, etc.,) from the great amount of easily degradable carbohydrates, which lead to a decrease in pH, followed by an ammonification process resulting in a rise of pH in compost (Kurola et al., 2011). The observed less pH fluctuation in MBM treatments than urea treatments can be explained by MBM nutrients being released slower than urea (Tibu et al., 2019).

The electrical conductivity value slightly fluctuated in both urea and MBM treatments. However, the values in MBM treatment were significantly lower than those in urea treatment throughout the composting time. An EC value below 4 mS·cm^−1^ is commonly regarded as suitable for safe growth of plants (García et al., 1991). Lower EC means greater maturity (Yang et al., 2021), indicating a better maturity in the MBM treatment than in the urea treatment.

Organic matter of the compost generally decreased over the composting period. The OM content dropped by 11.04 and 21.11 % in the urea-treated compost and MBM-treated compost, respectively. Decreasing trends of OM and TN were observed in both MBM and urea treatments, which is in accordance with a previous study (Feng et al., 2021) that demonstrated that OM and TN significantly decrease in the late-stage composting of rice straw and urea in an industrial-scale fermenter. Similarly, our results showed that both OM and TN decreased by the end of the composting, with lower TN in urea treatment than in MBM treatment. This is because N is largely bounded in MBM (Moller et al. 2015), resulting in lower bioavailability and poorer N supply than urea treatment. MBM compost recorded a lower OM content than urea treatment through composting. The nitrogen use efficiency of MBM was 80% or higher, while it was about 40% for urea (Jeng et al., 2007; Yang et al., 2011).

The germination index (GI) is a sensitive parameter to evaluate toxicity and the maturity of the compost (Selim et al., 2012). GI values of MBM and urea composts gradually elevated in the composting process. In the beginning, the GI values in both treatments were <30%. At the end of composting, the GI reached 95.11% in MBM treatment and 70.35% in the urea compost. The increased GI of MBM compost indicated a positive effect on the detoxification and nutrient availability in the final compost products. MBM has been reported as an organic fertilizer with increased available phosphorus for plants (Mondini et al., 2008; Möller, 2015).

Another parameter to evaluate the maturity of compost is the E_4_/E_6_ ratio, which represents the ratio of humic acid and fulvic acid in the compost. In this study, the E_4_/E_6_ ratio showed an overall decreasing trend with the composting time. The E_4_/E_6_ ratio in MBM composts was constantly lower than that in the urea compost but did not reach the significant level (Supplementary Figure S1). The nitrate–N (NO_3_-N) content of both treatments increased in the early stage of composting. The ammoniacal nitrogen (NH_4_-N) content of the compost generally decreased in the course of composting with an initial increase at the early phase of composting, where organic nitrogen was rapidly decomposed. The same trend was observed in MBM and urea treatments, indicating MBM has equal capability as urea in promoting transformations of organic nitrogen.

The lignin peroxidase (LiP) activity increased sharply, reaching at peak on day 14, and declined thereafter (Figure 2). Throughout the phases of composting (days 14–240), MBM treatment produced higher LiP than urea treatments, while on day 7, both the treatments produced statistically similar amounts of LiP. MBM treatment also superseded urea in cellulase activities, which reached at peak on day 30 and maintained thereafter. Lignocellulose degradation can be significantly improved by regulating the external environmental conditions during composting (Wei et al., 2019). The results of this study provide a clear difference in lignin degradation between MBM and urea treatments during composting of asparagus straw. It has been documented that lignocellulolytic activity depends on the types of lignocellulolytic microorganisms and their richness, as well as evenness of microbial communities, which were the primary driving factors of lignocellulose degradation (Kausar et al., 2013; Chen et al., 2021b). The addition of MBM increased soil microbial abundance and potential enzymatic activities, which might have stimulated the decomposition of recalcitrant soil organic matter (Jatana et al., 2020). MBM-amended compost recorded the highest values for ligninase activity on day 14 and for cellulase on day 30. Meanwhile, it was observed that the microbial community diversity and population in MBM treatment significantly increased within 14 days from the start of the experiment, suggesting MBM may stimulate a diverse microbial community, which is beneficial for lignocellulase enzyme secretion. Moreover, MBM provided a stable pH range through the composting process, which favored the enzyme activity and fits well with the optimal pH (5.91–6.24) for lignocellulose enzyme (Wang et al., 2011). MBM exerted significant stimulatory effects on enzymatic activity in this study, suggesting the potential of MBM application in the composting industry.

### Effect of meat and bone meal on microbial community profile in compost

**Figure 3:**
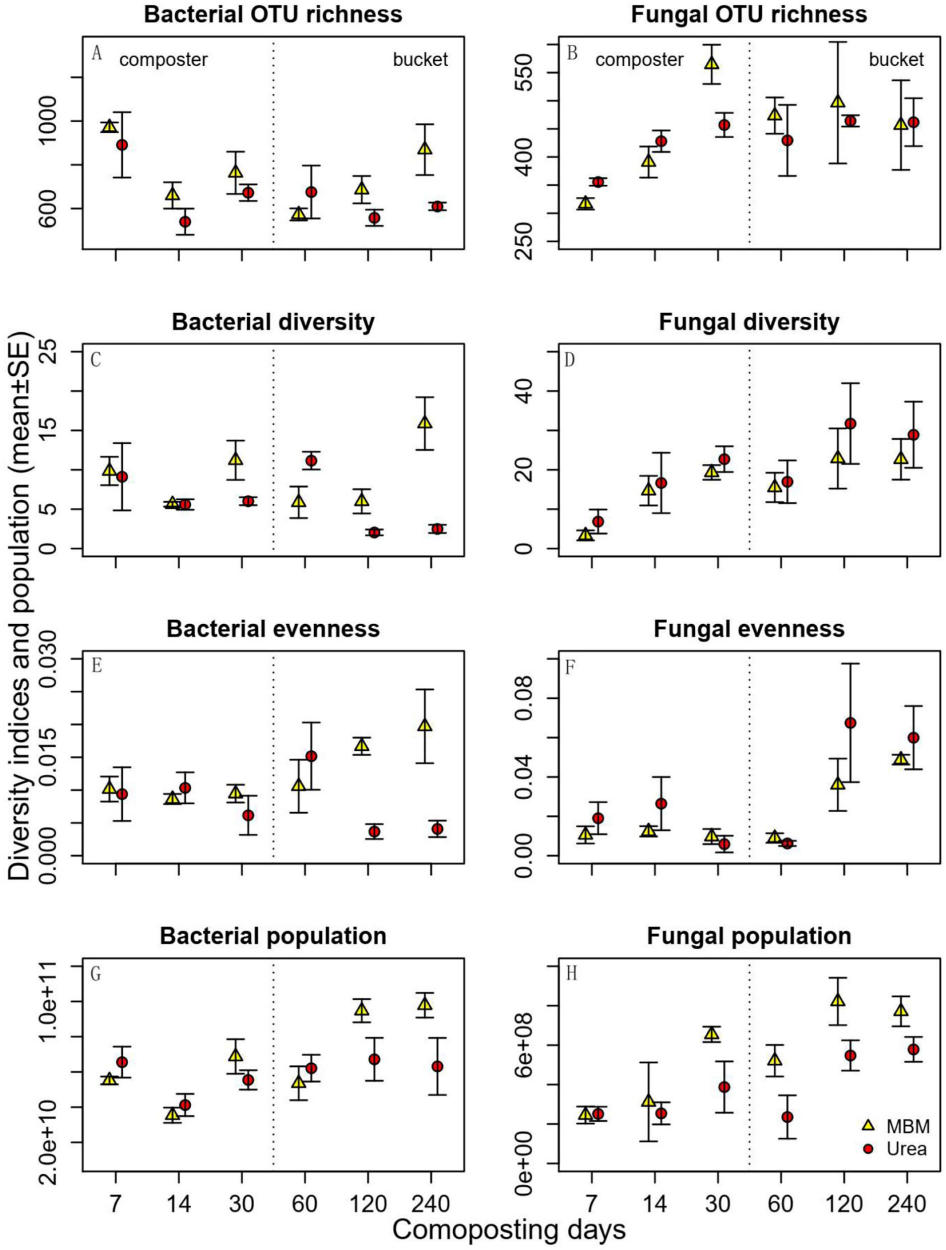
Predicted bacterial (left panels) and fungal (right panels) OTU richness, diversity, evenness, and population density (qPCR estimated ITS2 copy number per gram of dry soil) in the compost of meat and bone meal (MBM) and urea treatments (urea) and time (X axis). Error bars represent standard error.

In general, bacterial OTU richness, diversity, and evenness were higher in MBM-treated composts than in urea-treated ones, and these differences were more pronounced at the end of the experiment (Figures 3A,C,E). Bacterial OTU richness was higher in MBM-treated compost than in urea-treated compost. The differences were more pronounced on days 14 and 30, suggesting an increasing distinction between MBM- and urea-treated compost with time (Figure 3A). Bacterial richness was highest on day 7 in MBM-treated compost and decreased with time. Bacterial diversity and evenness responded likewise, with differences being more pronounced as composting time increased (Figures 3C,E). In this study, fungal OTU richness, diversity, and evenness showed a similar trend of being low in the beginning and reaching the highest level in the end of the experiment (Figures 3B,D,F). Fungal diversity and evenness were slightly higher in urea-treated compost than in MBM-treated compost. Fungal OTU richness was higher in the composters on day 30 in MBM treatment. Fungal evenness was greater in the buckets than in the composters, whereas it was similar across treatments in the composters. The microbial community diversity was impacted positively following the addition of MBM. This is consistent with a previous study dealing with the addition MBM as effective organic matter to soil to increase the size and activity of soil microorganisms (Mondini et al., 2008; Liu et al., 2019). Furthermore, it has been reported that MBM provides a greater amount of nutrients to activate complex and distinct microbial populations (Gryta et al., 2020; Haberecht et al., 2020).

**Figure 4:**
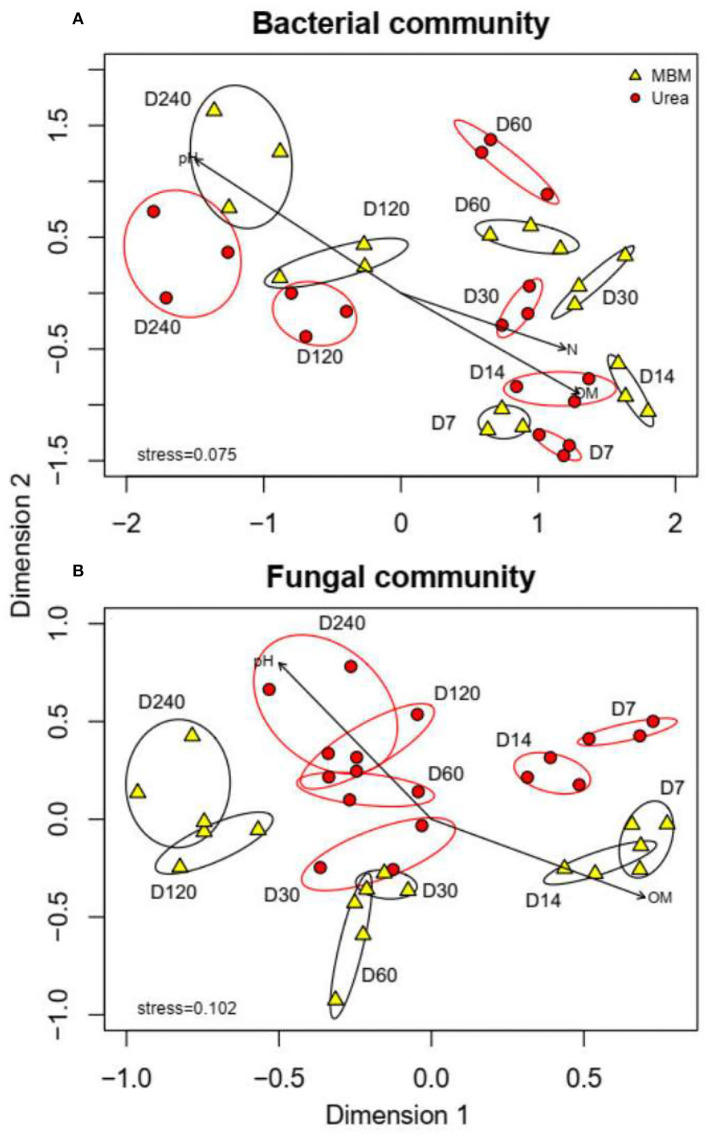
NMDS plots for bacterial and fungal communities of MBM treatments and urea treatments on days 7, 14, 30, 60, 120, and 240. MBM, meat and bone meal; urea, urea treatments.

The similarity of microbial communities between the MBM and urea treatments was assessed by NMDS analysis (Figure 4). The permutation analysis showed that at each sampling time (days 7, 14, 30, 60, 120, and 240), the bacterial and fungal community compositions between the MBM-treated and urea-treated composts were distinct (bacteria *r*^2^ = 0.467, *p* < 0.001, Figure 4A; fungal *r*^2^ = 0.522, *p* < 0.001, Figure 4B). The fungal community structure showed a significant difference between the MBM and urea treatments (*r*^2^ = 0.641, *p* < 0.001), while there was no significant difference between bacterial communities (*r*^2^ = 0.817, *p* = 0.063). The main driving factors of the fungal community structure were OM and pH, whereas OM, pH, and TN were the main driving factors for the bacterial community structure. Previous studies have found that temperature and organic matter were key factors affecting the distribution of microbial communities (Liu et al., 2018). The MBM and urea may influence the microbial communities of composts through their effect on temperature and pH of the pile, which is due to their different contents of nutrients and readily available forms of carbon and nitrogen (Mondini et al., 2008; Nogalska and Załuszniewska, 2021).

Bacterial and fungal population densities were estimated by qPCR, as the copy number of bacterial 16s rRNA or fungal ITS rRNA gene operons per gram of dry sample. We use this as a proxy of relative microbial abundances when comparing them between the MBM and urea treatments. Along with the composting process, the compost with MBM had a higher fungal population density than the urea treatment (Figures 3G,H). The increase in the bacterial population density by MBM was minor from days 7 to 120 but became pronounced in the final stages (days 120–240). The compost with MBM had a higher fungal population density than the urea-treated ones. Across sampling times, density was low in the beginning (day 7) and increased gradually thereafter. A previous study found that adding MBM biochar to petroleum hydrocarbon-contaminated soils can increase the total viable petroleum hydrocarbon-degrading populations (Karppinen et al., 2017). The increase in the microbial population density in the present work was not surprising due to the significant C, N, and P content of MBM.

**Figure 5:**
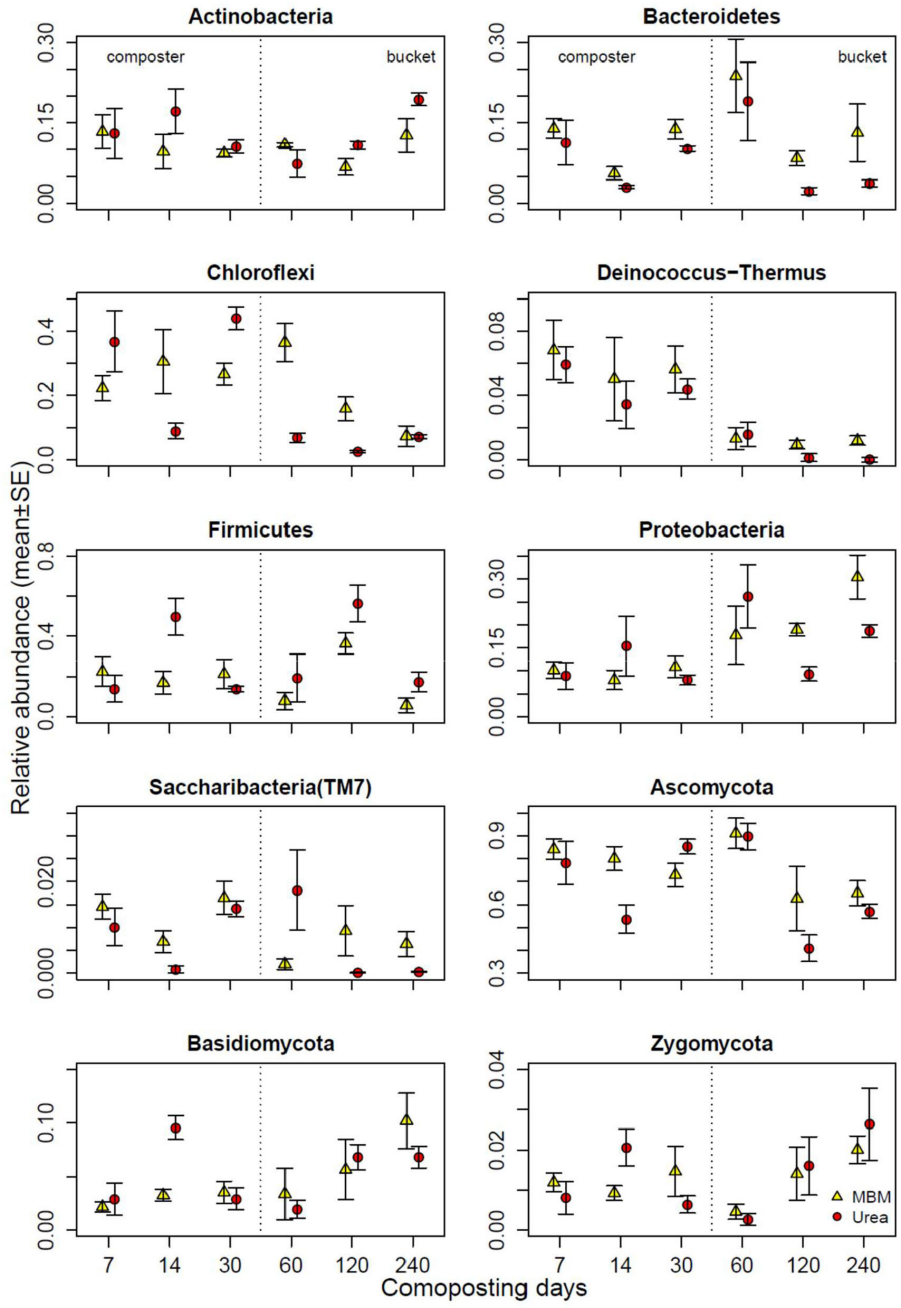
Relative abundances of bacterial and fungal phyla in compost of meat and bone meal (MBM) and urea treatments (urea) and time (X axis). Error bars represent standard error.

At the first sampling point (day 7), among all phyla identified in all composting samples, the predominant bacterial phyla were Firmicutes, Chloroflexi, Actinobacteria, Bacteroidetes, Proteobacteria, Deinococcus-Thermus, and Saccharibacteria (TM7), and the predominant fungal phyla were Ascomycota, Basidiomycota, and Zygomycota (Figure 5, Supplementary Figure S2). Firmicutes can grow at high temperatures and are widely distributed in the thermophilic phase of composting (Zhang et al., 2016). The abundance of all of the predominant phyla in MBM and urea treatments was similar, except Chloroflexi, which was 13% more abundant in urea composts than in the MBM compost. With the progression of composting, the abundance of Proteobacteria increased gradually and acquired the dominate place in the matured compost. Ascomycota accounted for more than 60% of the total fungal sequences on day 240. In the final product, MBM showed a pronounced influence on all of the predominant phyla, except Chloroflexi and Zygomycota. Compared with urea, the addition of MBM further increased the abundance of Bacteroidetes, Proteobacteria, Deinococcus-Thermus, Saccharibacteria, Ascomycota, and Basidiomycota on day 240 (Figure 5). The high content of organic matter of MBM suggests a potential positive effect on the microbiological properties of the compost. The microbial pool exerts a key role in determining the quality of the composted product (Mondini et al., 2008; Jatana et al., 2020). In this study, the MBM amendment leads to a clear variation in the bacterial and fungal community diversity and structure. This result was supported by a previous study, which showed that the application of MBM increased bacterial and fungal diversity and richness and shifted the soil bacterial community structure (Mondini et al., 2008). MBM presents a significant quantity of easily available substances, which can be readily utilized as the carbon and energy source by microorganisms (Mondini et al., 2008). These substances are mainly constituted by amino acids and polypeptides derived from partial hydrolysis of complex proteins during the thermal MBM treatment. Given that the microbial community composition and structure directly affect the microecological function and determine composting quality (Pan et al., 2021). MBM addition could be beneficial for asparagus straw composting in terms of microbial community diversity.

### Indicator taxa and suppression of plant pathogens by MBM addition

Indicator genera in MBM and urea treatments were identified (Table 1). A total of 11 bacterial indicator genera consist of one genus for the composters and 10 for the buckets, and 14 fungal indicator genera consist of four genera for the composters and 10 genera for the buckets. We found MBM reduced the sum relative abundance of pathogen genera *Phaeoacremonium, Acremonium*, and *Geosmithia* from 17.27 to 0.11%, which is consistent with the previous reports on the ability of MBM in reducing the abundance of plant pathogens in soils (Lazarovits et al., 1999). Notably, MBM promoted the relative abundance of *Trichoderma*, which has been reported as a disease-suppressive agent in composts and is a plant growth promoter (Cotxarrera et al., 2002; Stewart and Hill, 2014). One of the problems associated with the return of asparagus straw to field is that it often brings high risk of crop diseases, such as maize root rot, wheat common rot, and wheat sharp eyespot (Su et al., 2020). In our results, *Trichoderma* is enriched in the MBM treatment, suggesting a less expensive method to produce *Trichoderma*-rich compost products.

**Table 1:** Bacterial and fungal indicator genera in composters or buckets.

	**Stat**	***p***
**Bacterial genera**		
**In composter**		
**MBM**		
*Alterococcus*	0.851	0.015
**In bucket**		
**MBM**		
*Microbulbifer*	0.993	0.009
*Rhodothermus*	0.939	0.022
*Phycisphaera*	0.921	0.029
*Alterococcus*	0.915	0.023
*Bradymonas*	0.885	0.01
*Roseiflexus*	0.823	0.033
**Urea**		
*Caldalkalibacillus*	0.964	0.019
*Streptomonospora*	0.87	0.03
*Kribbella*	0.876	0.015
*Syntrophomonas*	0.745	0.039
**Fungal genera**		
**In composter**		
**MBM**		
*Trichoderma*	0.917	0.024
**Urea**		
*Ampelomyces*	0.909	0.034
*Madurella*	0.791	0.028
*Sagenomella*	0.789	0.026
**In bucket**		
**MBM**		
*Myriococcum*	0.942	0.011
*Kernia*	0.745	0.026
*Remersonia*	0.942	0.023
*Chrysosporium*	0.892	0.043
*Phanerochaete*	0.874	0.022
*Sagenomella*	0.745	0.024
*Zopfiella*	0.745	0.024
**Urea**		
*Phaeoacremonium*	0.996	0.042
*Acremonium*	0.931	0.02
*Geosmithia*	0.815	0.033

MBM, meat and bone meal; Urea, urea treatments.

Thus far, *Trichoderma* spp. is among the most studied fungal plant pathogen biocontrol agent. Depending on the strain, the use of *Trichoderma* in agriculture can provide numerous advantages in control of pathogenic and competitive/deleterious microflora by using a variety of mechanisms and improvement of the plant health and stimulation of root growth (Vinale et al., 2008). To achieve these advantages, *Trichoderma harzianum* inoculant is added to well-matured compost (Siddiqui et al., 2008; Organo et al., 2022), and the results suggest that *Trichoderma* spp. growth can be promoted by MBM, thus suggesting a less expensive method to produce *Trichoderma* spp.-enriched compost products. The mechanisms of action used by *Trichoderma* (competition, antibiosis, parasitism, and systemic-induced resistance) are influenced by the concentration and availability of nutrients (carbohydrates in lignocellulosic substances, chitin, lipids, etc.,) within the organic matter. Meat and bone meal serves as an easily degradable C and N source due to the mandatory heat treatment (heating with steam vapor at 133°C for 20 min under a pressure of 0.3 Mpa) that MBM must undergo (EC, 2002). Thus, MBM straw co-composts represent an optimal substrate for *Trichoderma* spp., thus encouraging their establishment into the compost products. One of the future objectives of this study is to elaborate a better understanding of the activities of *Trichoderma* strains in MBM straw composting, leading to selection of strains suitable for organic farming, which is becoming increasingly popular worldwide.

### Practical application and future perspectives

This study was conducted in 220 L, and the results of meat and bone meal as an additive were reliable. The mechanism analysis made with the microbial community reflected the microbial process in whole composting process. Therefore, the results can be extended to industrial scale. In addition, the MBM-assisted composting of straws can become a sustainable and economically feasible strategy to recycle meat and bone meal. It can be assumed that alternatives of reusing MBM other than disposal in landfill could probably be envisaged. A better understanding of the principles regulating the interaction between fungal pathogens and their suppressive agents such as *Trichoderma* in MBM straw composting would enhance the practical application of MBM. Further study is needed to identify the complex interactions that beneficial microbes established against pathogens. A better understanding of these processes and of the molecular cross-talk causing the suppression will result in the application of safer and less expensive methods to utilize MBM.

## Conclusion

The physicochemical characteristics and microbial properties of the compost samples were measured for 240 days. Compared to additive urea, adding MBM to asparagus straw compost stabilized pH, extended the thermophilic phase, and increased the germination index and enzymatic activities. MBM treatments had higher microbial community diversity and population density. In the phylum level, MBM addition increased the relative abundance of Chloroflexi in the thermophilic phase and Proteobacteria in cooling and maturation phases. In the genus level, MBM improved organic matter-degrading bacteria (*Alterococcus*) and lignocellulose-degrading and disease-suppressing fungi (*Trichoderma*), while inhibited plant pathogenic fungi (*Phaeoacremonium, Geosmithia*, and *Acremonium*), suggesting MBM addition improved asparagus straw composting efficiency and safety.

## Data availability statement

The datasets presented in this study can be found in online repositories. The names of the repository/repositories and accession number(s) can be found below: https://www.ncbi.nlm.nih.gov/genbank/, SAMN20518185–SAMN20518220; SAMN20518136–SAMN20518171.

## Author contributions

XLiu: data analyses and writing—original draft preparation. XLi: lab work and writing. YH: resources and investigation. AS and MR: revision. YL: data analyses. QW: resources and investigation. NH: conceptualization, methodology, supervision, and software. All authors contributed to the article and approved the submitted version.

## Funding

This study was financially supported by the National Natural Science Foundation of China Youth Fund (project number: 42007048), the Science and Technology Commission of Shanghai Municipality (Project number: 22230713300) and Shanghai Pujiang Project (project number: 20PJ1405300).

## Conflict of interest

Author YH is employed by Pudong Development Group Co., Ltd. Author QW is employed by Boda Environmental Protection Co., Ltd. The remaining authors declare that the research was conducted in the absence of any commercial or financial relationships that could be construed as a potential conflict of interest.

## Publisher's note

All claims expressed in this article are solely those of the authors and do not necessarily represent those of their affiliated organizations, or those of the publisher, the editors and the reviewers. Any product that may be evaluated in this article, or claim that may be made by its manufacturer, is not guaranteed or endorsed by the publisher.
