# Supplementary material for: Meat and bone meal stimulates microbial diversity and suppresses plant pathogens in asparagus straw composting
Source: Front Microbiol. 2022 Sep 20;13:953783. doi: 10.3389/fmicb.2022.953783 (PMC9530395; doi:10.3389/fmicb.2022.953783)

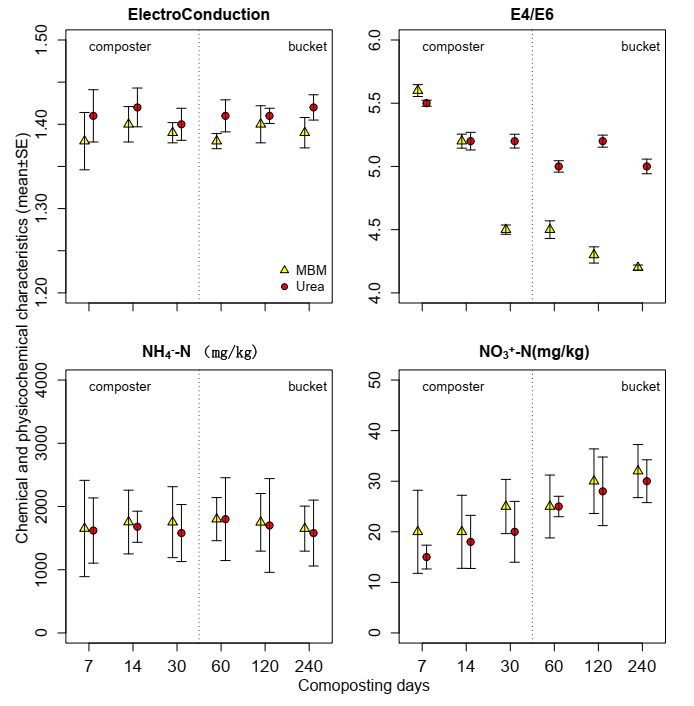
**Supplementary Fig. S1.** Dynamics of electro conduction, E4/E6, NH^4^-N, NO_3_-N during composting process. MBM – asparagus straw with additive meat and bone meal, urea– asparagus straw with additive urea. Error bars are standard deviations of the means of triplicates.


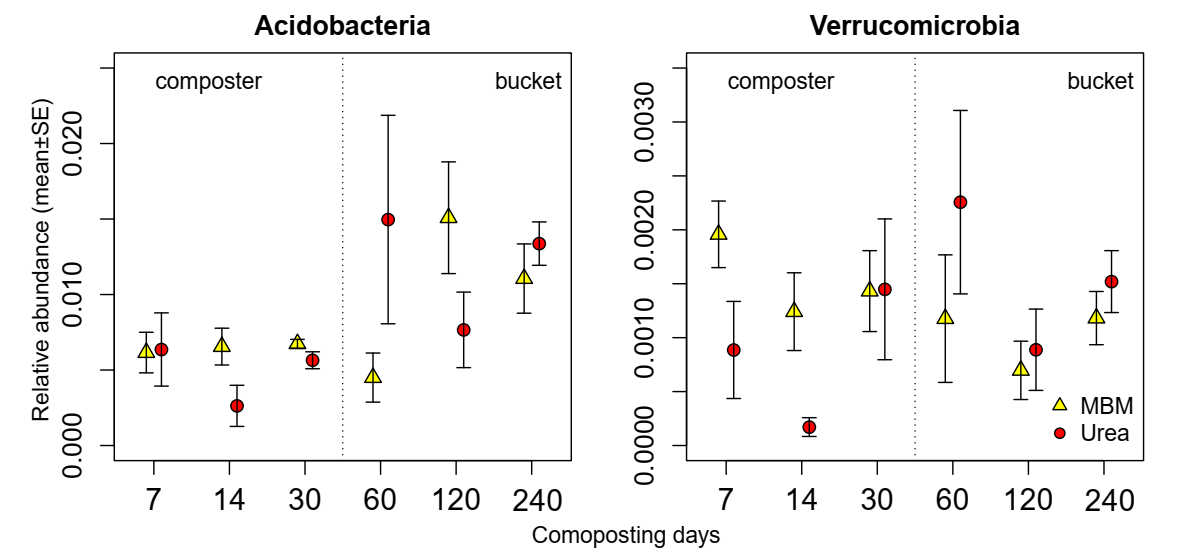
**Supplementary Fig. S2** Relative abundances of bacterial phyla in compost of meat and bone meal (MBM) and urea treatments (symbols) and time (X axis). Error bars represent standard error.

Highlights :

- Co-composting of asparagus straw and meat and bone meal (MBM) in 220L composters
- 15% MBM composters reach over 60℃ similarly to composters with 2.5% urea
- MBM stabilizes pH and prolongs thermophilic phase as compared to urea
- Microbial diversity is greater in MBM treatments than in urea ones
- MBM additions suppress plant pathogens in compost products


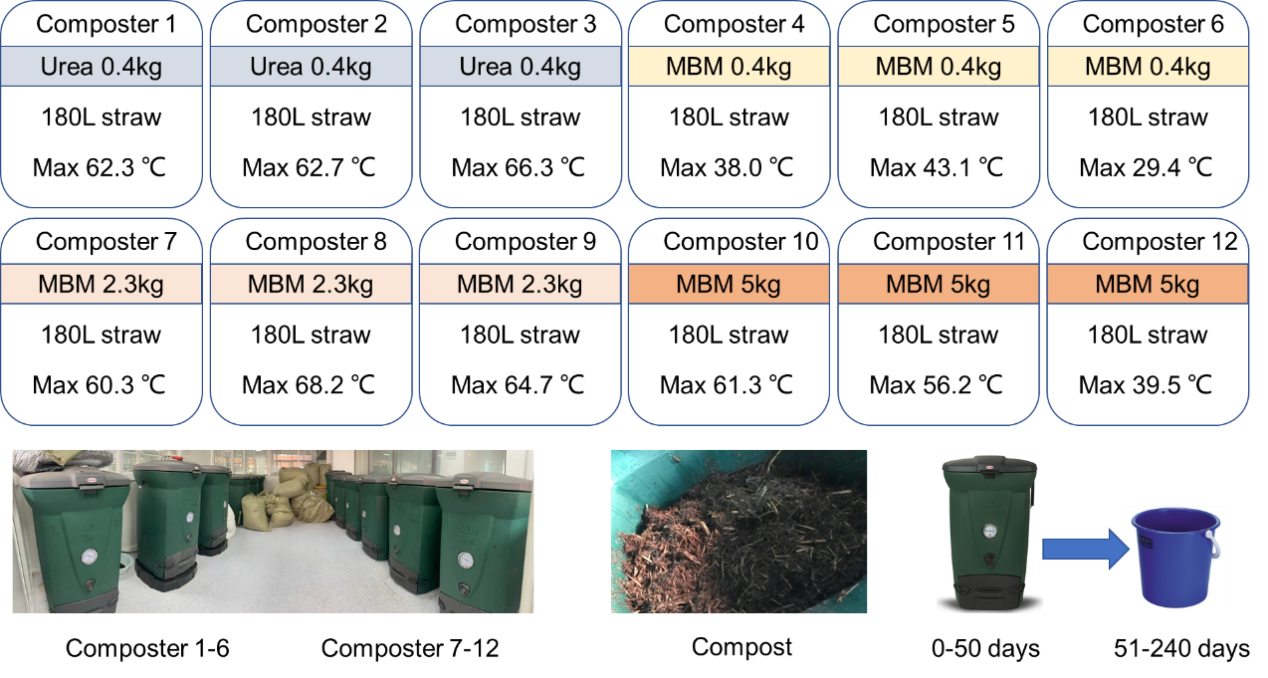

Supplement: Supplementary file 1 [file Data_Sheet_1.docx]
